# Supplementary material for: Cross-Oxygen Gradients Transcriptomic Comparison Revealed the Central Role of MAPK and Hippo in Hypoxia-Mediated Mammary Proliferation Inhibition
Source: Antioxidants (Basel). 2024 Feb 26;13(3):288. doi: 10.3390/antiox13030288 (PMC10967332; doi:10.3390/antiox13030288)
Supplement: Supplementary file 1 [file antioxidants-13-00288-s001.zip › Supplementary Material.pdf]

Supplementary Table S1: Summary of Illumina RNA-seq data.

Sample	Raw reads	Raw bases	Clean reads	Clean_bases	Q20 (%)	Q30 (%)	GC (%)	N (ppm)
1%_1	44988290	6748243500	44353624	5976491644	98.54	95.68	54.38	3.51
1%_2	41694406	6254160900	41641178	6191731297	97.73	93.59	54.32	0.84
1%_3	46727690	7009153500	46663404	6936762037	97.93	94.14	53.82	2.32
1%_4	44535946	6680391900	44475872	6603544785	97.97	94.16	54.59	2.54
1%_5	41592210	6238831500	41536364	6168193546	97.97	94.14	54.25	0.84
6%_1	44836176	6725426400	44768180	6649341273	97.93	94.14	53.96	2.28
6%_2	43627040	6544056000	43569172	6477009837	97.84	93.91	53.93	2.34
6%_3	44191494	6628724100	44133328	6560148446	97.98	94.24	54.5	2.24
6%_4	47061174	7059176100	46991674	6980637363	97.83	93.89	53.82	2.46
6%_5	47320888	7098133200	47250416	7019344731	97.68	93.55	54.03	2.44
11%_1	39225836	5883875400	39165282	5816785401	97.72	93.64	53.8	2.49
11%_2	45982940	6897441000	45089224	5882843593	98.71	96.07	54.21	3.73
11%_3	44534824	6680223600	44474510	6608840937	97.93	94.09	54.05	2.17
11%_4	44076444	6611466600	44017752	6549849946	97.93	94.09	53.73	2.08
11%_5	44729522	6709428300	44668632	6643805632	98.13	94.58	54.04	2.15
16%_1	44322480	6648372000	44266424	6587841024	97.94	94.13	53.55	2.2
16%_2	41408870	6211330500	41352410	6145694267	97.93	94.09	53.77	2.26
16%_3	41779038	6266855700	41725744	6208170755	97.61	93.37	53.46	2.32
16%_4	44558888	6683833200	44502788	6618852386	97.99	94.19	54.02	2.28

16%_5	44521710	6678256500	44310402	6368254017	98.28	95	54.51	3.31
21%_1	44858278	6728741700	44794078	6658511851	97.71	93.61	53.63	2.49
21%_2	45457284	6818592600	45160424	6616365963	98.01	94.43	52.65	2.74
21%_3	41047596	6157139400	40988212	6090135193	97.7	93.59	53.66	2.53
21%_4	42065806	6309870900	42002270	6234814011	97.98	94.22	53.72	2.56
21%_5	37926128	5688919200	37866830	5616226147	97.77	93.76	53.92	2.46

Supplementary Table S8: The primer sequences of NGFR, CSF1, KDR, IL1R1, PPP2R2B and β-actin for real-time quantitative PCR.

Gene	Primer	Sequence (5'-3')	Size(bp)
NGFR	Forward	TTCTCGGACGTGGTGAGCGC	217
	Reverse	CGGTGTTCTGCTTGTCCTGGC	
CSF1	Forward	CTGCGAGCCCCAGTCACACC	217
	Reverse	GGGTGCAGTGCTGGGGTCTT	
KDR	Forward	CAGCCCACCCTCACTGCCTT	293
	Reverse	TTCTCCTGCTCGGTGGGCTG	
IL1R1	Forward	AGCTGTTCATCTGTGGCCGAGA	137
	Reverse	TGAATTCCCCGGCCAGCTGA	
PPP2R2B	Forward	TCCAGAGCCATGAACCCGAGT	216
	Reverse	TGGTGGCAGGATCTCGCAGC	
β-actin	Forward	CATGTGCAAGGCCGGCTTCG	113
	Reverse	ACGAGTCCTTCTGGCCCATGC	

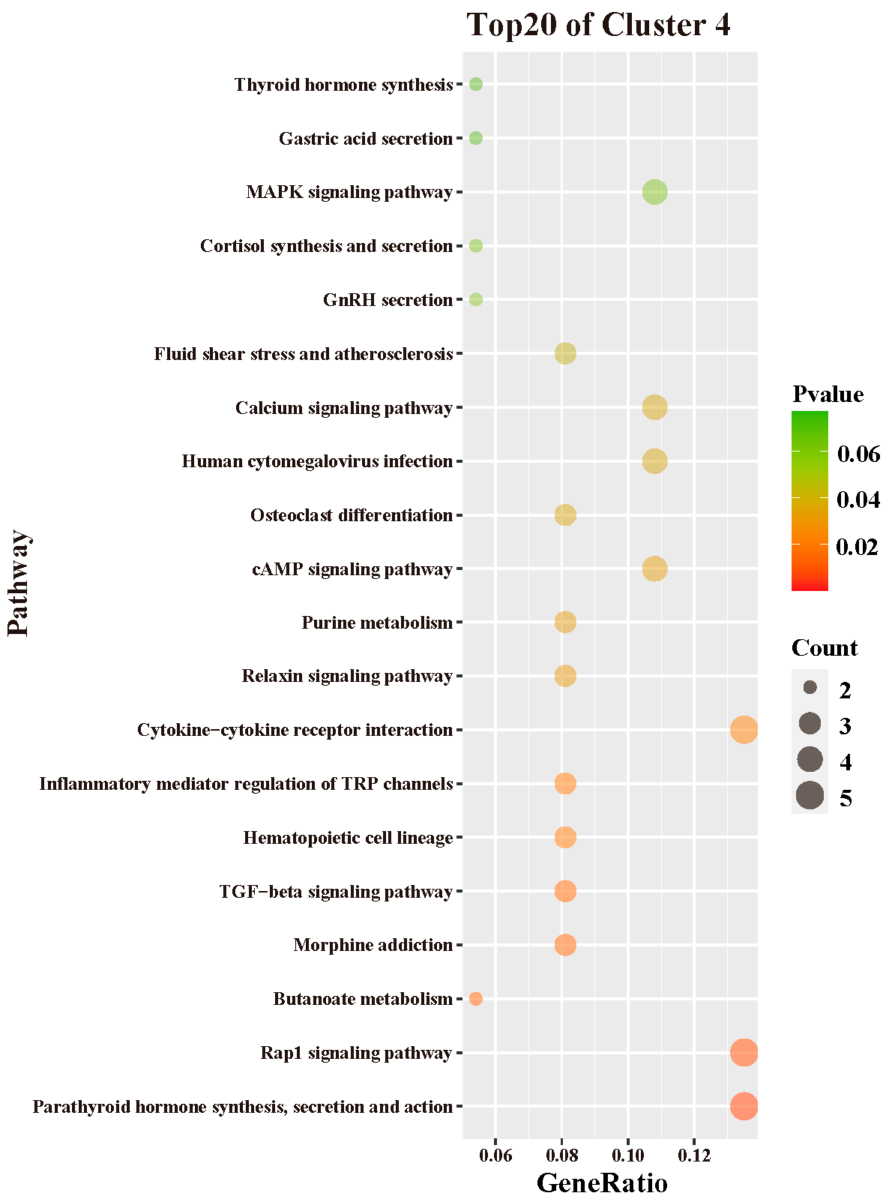

Supplementary Figure S1: KEGG pathway enrichment analysis of Cluster 4. Bubble charts represent the top 20 significantly enriched KEGG pathways.

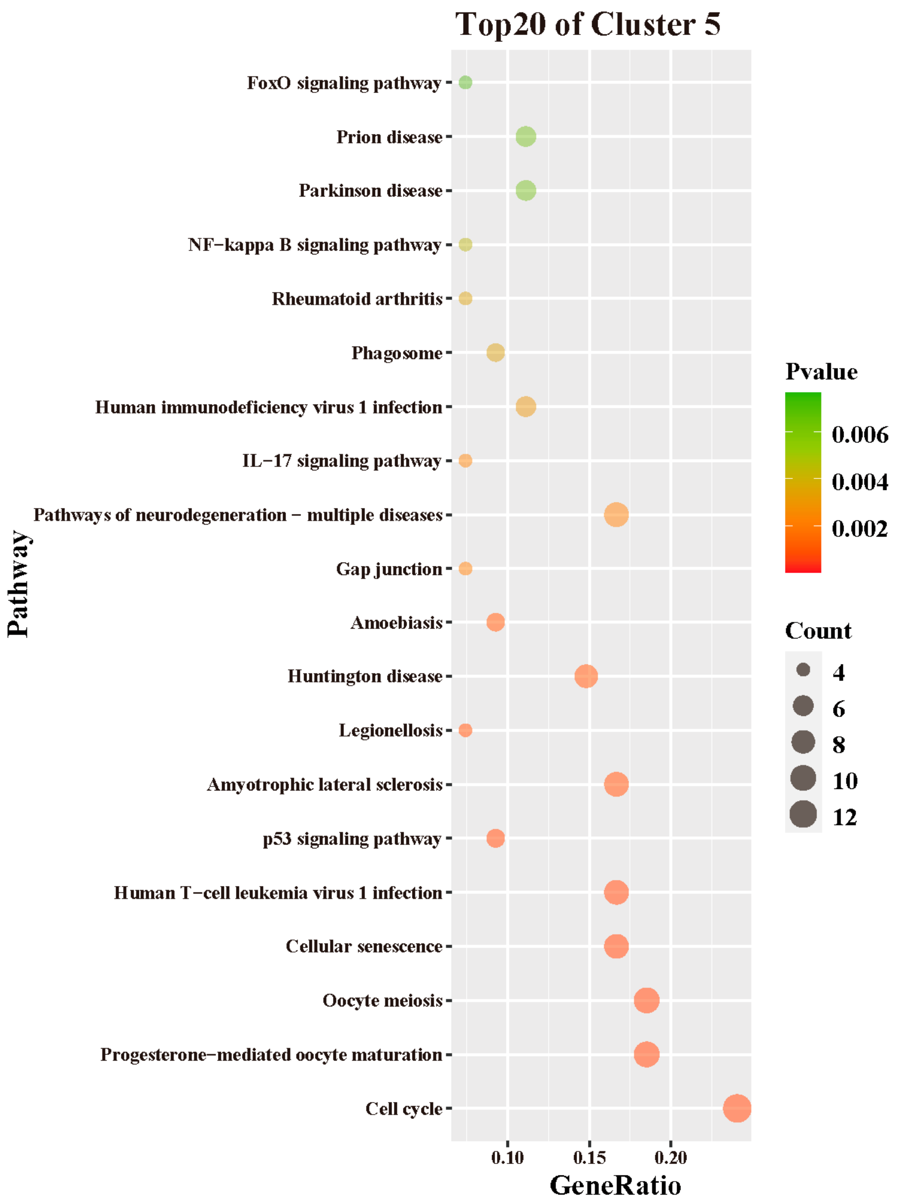

Supplementary Figure S2: KEGG pathway enrichment analysis of Cluster 5. Bubble charts represent the top 20 significantly enriched KEGG pathways.

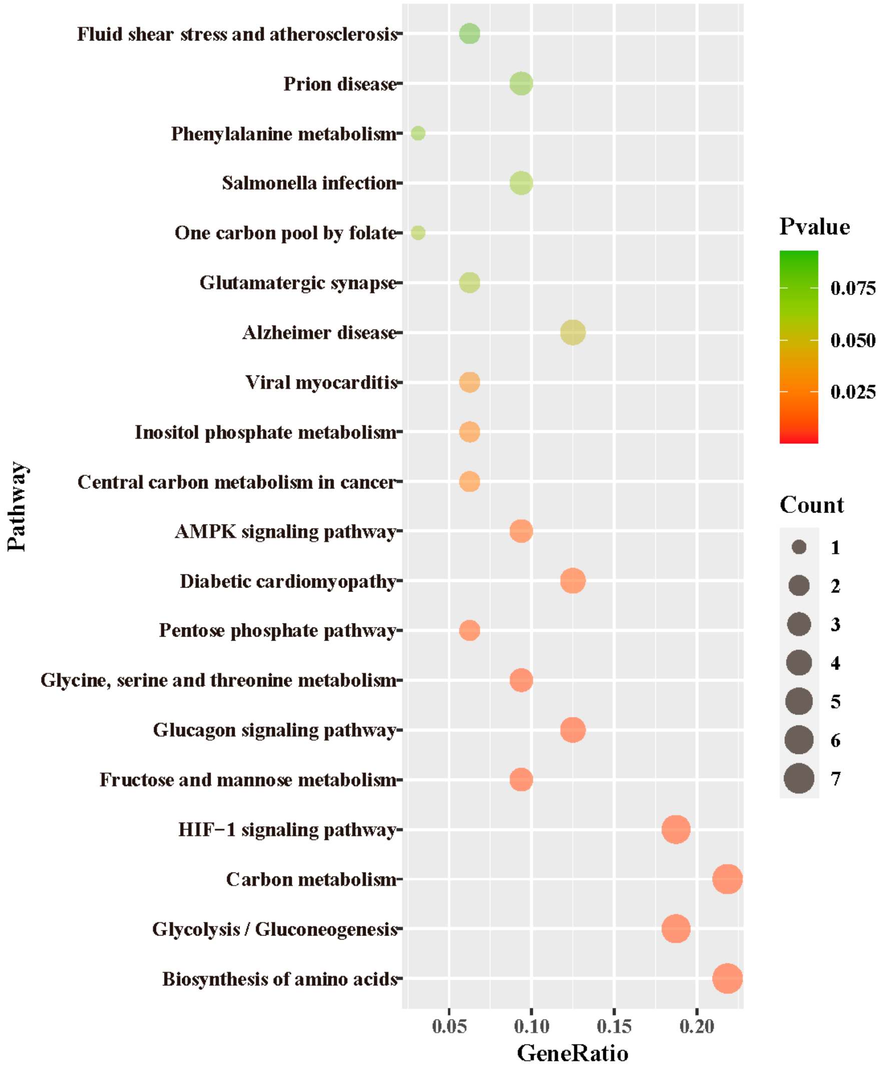

Supplementary Figure S3: KEGG pathway enrichment analysis of Cluster 8. Bubble charts represent the top 20 significantly enriched KEGG pathways.

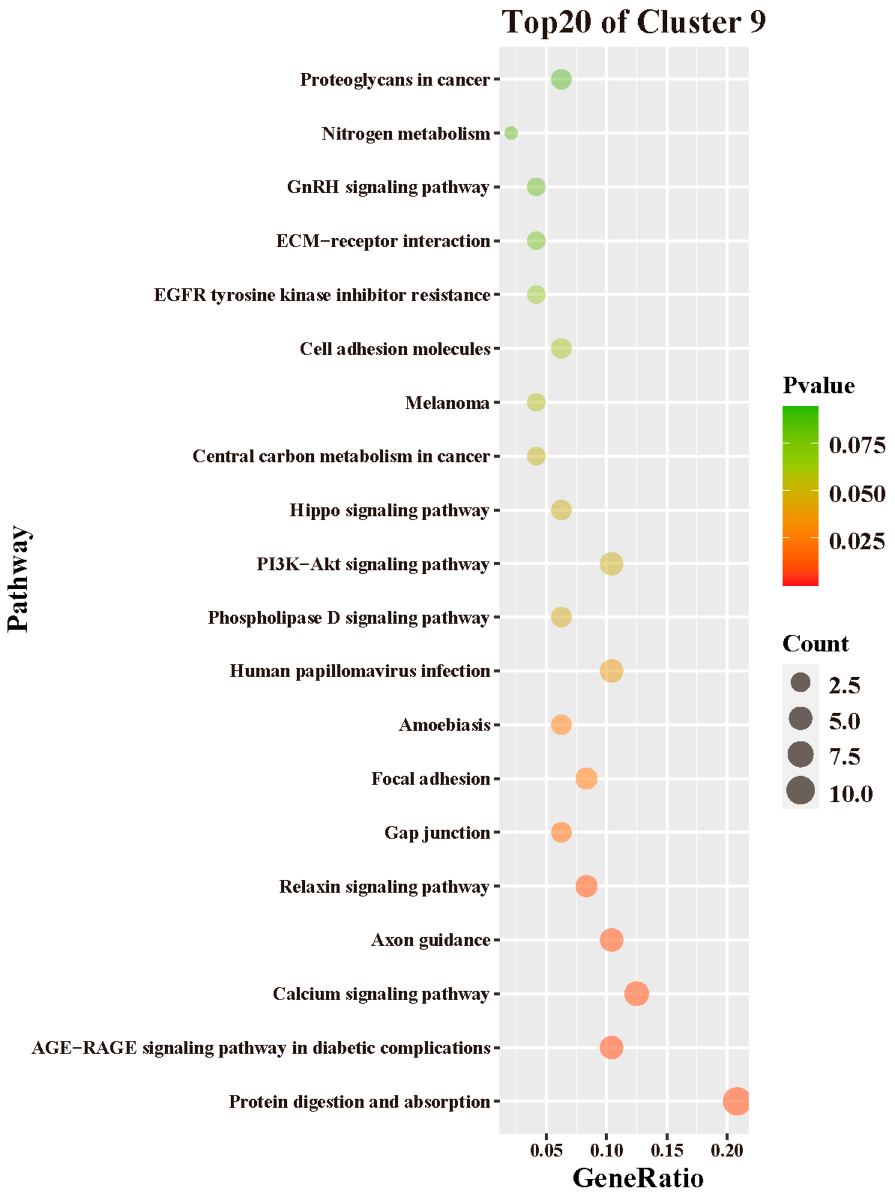

Supplementary Figure S4: KEGG pathway enrichment analysis of Cluster 9. Bubble charts represent the top 20 significantly enriched KEGG pathways.

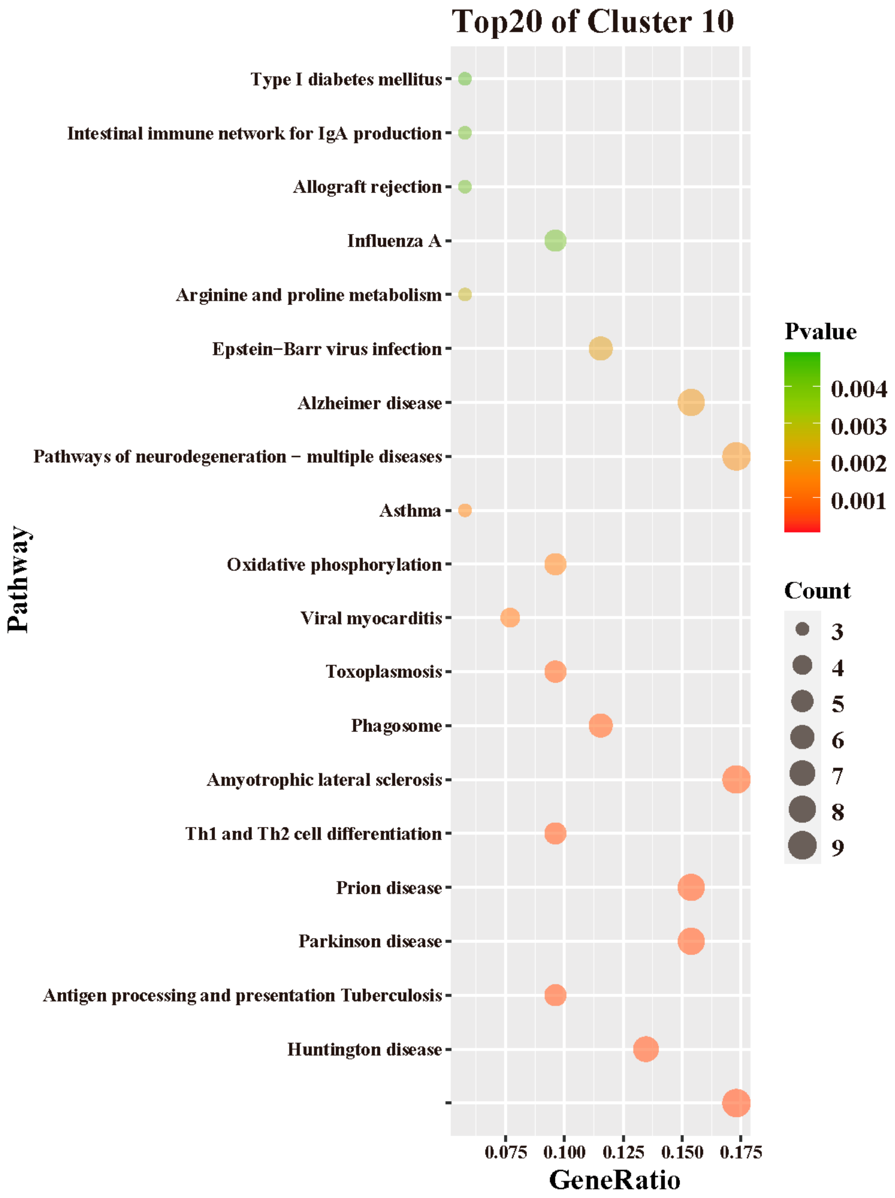

Supplementary Figure S5: KEGG pathway enrichment analysis of Cluster 10. Bubble charts represent the top 20 significantly enriched KEGG pathways.
